# Knowledge, preferences and willingness to use at-home prostate and colorectal cancer screening tests in African American and Haitian men

**DOI:** 10.3332/ecancer.2021.1310

**Published:** 2021-10-25

**Authors:** Alexandra Jean-Louis, Fern J Webb

**Affiliations:** 1 Department of Health Sciences, College of Health Professions and Sciences, University of Central Florida, Orlando, FL 32816, USA; 2 Department of Community Health and Family Medicine (Jacksonville), College of Medicine, University of Florida, Jacksonville 32209, USA

**Keywords:** prostate cancer, colorectal cancer, cancer screening, at-home screening tests, African American, Black, Haitian

## Abstract

Haitian (HA) and African American (AA) men have the highest prostate cancer (PCa) and colorectal cancer (CRC) age-adjusted mortality rates compared with other racial/ethnic groups worldwide. One contributing factor to mortality differences is that a low percentage of age-eligible HA and AA men screen for PCa and CRC, even when healthcare access and insurance are available. Reasons for cancer screening disparities may be differences in knowledge, preferences and willingness in HA and AA men. However, limited information exists on whether HA and AA men are knowledgeable about and are willing to be screened for PCa and CRC. Moreover, understanding preferences and willingness of HA and AA men to use cancer screening tests completed at home is of paramount importance given the current pandemic. We used a cross-sectional study design to assess HA and AA men’s knowledge, preferences and willingness to use at-home PCa and CRC screening tests. Survey items were developed from existing surveys assessing CRC knowledge and willingness to screen. Institutional Review Board approval was obtained to invite persons who identified as male, at least 18 years of age and Black (as either AA and/or HA) to complete the survey. A total of 36 Black men completed the survey; 42% self-identified as both ‘African American’ and ‘Haitian’ (AA/HA), 44% identified only as AA, and 14% identified only as HA. Regardless of race or ethnicity, 75% of all participants were 45 years or younger (range: 18–85). Although more than 80% of all participants heard about PCa and CRC, only 50% of participants aged at least 50 years old were screened for CRC. The majority of participants (AA/HA = 67%; HA = 80%; AA = 56%) were unaware of at-home CRC screening tests; however, 80% of AA/HA men and 60% of HA men were willing to use an at-home CRC screening test compared to 44% of AA men.

## Background

Black men living in the United States (U.S.) have high incidence and mortality due to prostate cancer (PCa) and colorectal cancer (CRC). PCa, the most commonly diagnosed cancer in Black men [1], is a malignancy in the prostate gland [2]. CRC refers to malignancies or uncontrolled cell growth in the colon, bowel or rectum [3].

The ‘Black’ population comprises many ethnic groups, including men identifying as Haitian (HA) or African American (AA) [4] who disproportionately develop and die from PCa and CRC [1–3]. In fact, PCa incidence and mortality among HA men living in the U.S. is among the highest worldwide; 767 per 100,000 (10^5^) HA men are diagnosed with PCa with an age-adjusted mortality rate of 403 per 10^5^ HA men [2]. Regarding AA men, PCa incidence is almost 60% higher than the PCa incidence experienced by Caucasian men [5]. The PCa mortality rate among AA men is more than two times greater than the PCa mortality rate in Caucasian men [5]. Although a 2016 analysis by Pinheiro *et al* [6] showed that HA men have lower PCa mortality compared to US-born Black men, PCa and CRC mortality among HA and AA men are significantly higher than other racial and ethnic groups.

## CRC screening methods

There are two types of CRC screening options, visual exams and stool-based tests [1, 3]. Visual exams are invasive procedures which use computed tomography colonography, colonoscopy and flexible sigmoidoscopy. On the other hand, stool-based tests provide a non-invasive alternative for CRC examinations that check the stool for indications of blood, CRC or polyp cells [1, 3]. The advantages of stool-based tests are the inexpensiveness and capability to acquire samples from home [1, 3]. The types of stool-based tests currently available are the faecal immunochemical test, stool DNA tests and Guaiac-based faecal occult blood test (gFOBT).

## PCa screening, diagnostic and risk-assessment methods

The diagnostic tests for PCa are performed in the absence of detectable symptoms or indications of disease [5, 7]. Screening, diagnostic and risk-assessment methods available include the prostate-specific antigen blood test transrectal ultrasound guided biopsy, prostate urine risk (PUR) test and digital rectal exam [5, 7]. With the exception of the PUR test, the previously listed examinations are invasive.

### PCa and CRC screening among HA and AA men

One major contributing factor for higher cancer mortality among HA and AA men is failure to screen for PCa and CRC at the same rates as other racial or ethnic men, resulting in significant screening disparities. HA men screen less for PCa and CRC when compared to US-born Black men [8]. Moreover, May *et al* [9] estimated that 20% of all CRC deaths in Black men could be avoided with increased CRC screening. Reasons cited for low screening have traditionally been limited healthcare access and/or coverage. However, recent research by Roberts *et al* [10] found that PCa screening was not related to healthcare access or insurance status among the 264 Black men within the age for recommended cancer screening in their study.

Given the variety of screening modalities for PCa and CRC, researchers have investigated what Black males mostly prefer. Palmer *et al* [11] provided insight on personal CRC screening preferences in a study with 60 AA men. The researchers discovered that AA men preferred the FOBT, perceiving it as non-invasive and of low cost. Francois *et al* [12] found similar results among HA immigrant men residing in Brooklyn, New York. Male study participants admitted to being more comfortable with the FOBT because it can be done at home; it is not as risky as a colonoscopy procedure (i.e. fear of dying), and a general unawareness of colonoscopy tests.

There is limited information on whether HA and AA men, starting as young as 25 years old, are knowledgeable about, and willing to be screened for PCa and CRC. To date, very few studies have investigated the specific PCa screening tests preferred by HA and AA men which is important for physicians to follow U.S. Preventive Services Task Force recommendations to initiate individualised PCa screening care adapted to the preferences of each patient [13]. Understanding preferences and willingness of HA and AA men to use cancer screening tests completed at home is important. The current pandemic has significantly impacted how relatively healthy individuals obtain medical care. Thus, discovering alternative approaches for screening populations at increased risk for high PCa and CRC mortality is of paramount importance. Even less is known regarding HA and AA men’s preferences to perform and receive PCa and CRC at-home testing information. The purpose of this research was to examine knowledge, willingness and preferences to perform at-home PCa and CRC screenings in Black adult men, as well as report barriers and facilitators to use at-home screening tests.

## Methods

The cross-sectional study was designed to investigate willingness of HA and AA men to perform at-home cancer screening tests provided they exist (i.e. prostate (PCa); colorectal (CRC)); report participants’ awareness of at-home CRC screening tests currently available for use and identify reasons that prevent or promote use of at-home cancer screenings.

Population of focus: The University of Florida’s Institutional Review Board (IRB) provided approval to conduct this research. The focus population were males, at least 18 years old, who self-identify as HA and/or AA.

Research design and sampling: Sample size & power: A total of 32 men were required to detect a large effect size (0.50) between two groups (AA men and HA men; degree of freedoms = 2), using an alpha of 0.05 with a power of 0.80. Accounting for 5% missing information, 36 men were desired to examine specific aims.

Eligibility: Individuals who opened the survey were unidentified and had to pass preliminary screening questions on Qualtrics, an online platform used to create surveys and analyse responses. Each participant was asked for their age and if they identified as a HA, HA and AA, or AA man. If ‘yes’ was selected, participants were directed to the informed consent form. To begin answering the survey questions, ‘I agree to participate’ had to be chosen by each respondent after reading the informed consent form.

Recruitment approach: Our approach was to conduct outreach in venues traditionally known to have a high representation of AA and/or HA men. Recruitment began by informing family and friends who are mostly members of AA and HA groups. The survey was also posted on IRB-approved websites. The researcher also employed snow-ball sampling, where individuals who completed the survey were encouraged to share with others who might be interested and eligible.

Survey tool: Survey questions were modelled from Francois et al’s [12] ‘Attitudes and beliefs about colon cancer screening’ instrument to fit this study’s context. Questions, listed in Table 1, were specifically designed to assess knowledge about PCa and CRC, knowledge about CRC at-home screening tests, willingness to participate in PCa and CRC screening, barriers for not being able to screen and preferences for receiving PCa and/or CRC information.

*Knowledge* regarding PCa and CRC was assessed using the following question: ‘Do you know what prostate and colon cancer are?’ Participants’ knowledge about CRC at-home screening tests was evaluated using the following question: ‘There are colon cancer screening tests that can be done at home’. A composite knowledge variable was created where ‘Yes’ responses to either of these two questions were coded as 1 and ‘No’ responses were coded as 0.

*Current testing* was assessed by asking: ‘Have you ever tested for prostate and colon cancer?’ A composite testing variable was created where ‘Yes’ responses were coded as 1 and ‘No’ responses were coded as 0.

*Willingness to screen* was assessed by asking ‘If a prostate cancer screening test that can be done at home were made would you buy and use it?’ and ‘If a colon cancer screening test that can be done at home were made would you buy and use it?’ A composite willingness variable was created where ‘Yes’ responses to either of these two questions were coded as 1 and ‘No’ responses were coded as 0.

*Barriers* were assessed by asking ‘If any, select what will stop you from doing a cancer test correctly at home? Select all that apply’ with the option to include additional responses.

*Preferences* on what would help to complete a cancer screening test at home were assessed by asking: ‘What would help you do a cancer test correctly at home? Select all that apply’.

Survey distribution: The distribution of the survey occurred exclusively online. The survey was self-administered and completed electronically using Qualtrics.

**Figure 1.**
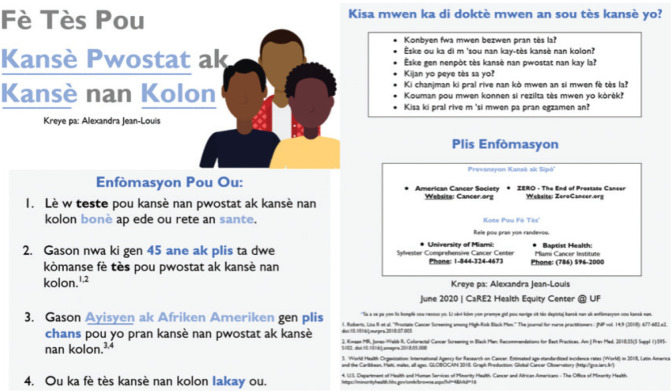
The resource guide displayed in English and Haitian-Creole after the completion of the online survey.

Resource guide: At the end of the survey, a resource guide was provided that included facts about PCa and colon cancer. As well as questions that patients can ask their providers, online cancer sources and locations to obtain cancer testing and treatment. The resource guide was available in English and Haitian-Creole (see Figure 1); to ensure consistency, the resource guides were translated into Haitian-Creole by two persons whose first language is Haitian-Creole. The resource guide was then back-translated into English by two different persons to ensure that the Haitian-Creole translation was consistent with the original English edition. Participants could either save the resource guide by 1) downloading it to their personal devices and/or 2) taking a screenshot.

### Analysis

All responses were downloaded from Qualtrics and analysed using SAS v. 9.4.

Descriptive statistics were conducted to show frequencies for categorical responses, and mean scores for continuous variables. Chi-square statistics were used to test for statistical significance at the 0.05 level. Responses to knowledge and previous testing are reported by race and ethnicity to include men who responded as AA, HA or AA/HA. We also dichotomised age into two categories: >45 and 45 when examining screening experiences given that current recommendations are for men at-risk for PCa or CRC should begin screening at 45 per American Cancer Society (ACS) recommendations [14].

Analytic statistics were conducted using logistic regression modelling where knowledge, testing and willingness were separately entered as outcome variables, and ethnicity and age were entered as covariates. Odds ratio (OR) and 95% confidence intervals (CIs) were tabulated in logistic regression models. We added 0.5 to each cell following the Haldane [15] and Anscombe [16] correction methods to account for zero cell values.

## Results

A total of 36 men completed the survey. Of the 36 men who completed the survey, 16 (44%) identified as AA, 5 (14%) identified as HA and 15 (42%) identified as both AA and HA (AA/HA). As shown in Table 2, 27 (75% of) men, regardless of race or ethnicity, were 45 years old or younger (range: 18–85). When examining screening knowledge, 100% of men > 45 years old were aware of PCa and CRC, compared to 67% of younger men who have heard about PCa and CRC (Table 2). However, an additional 19% of younger men reported only hearing about PCa, resulting in 86% of all younger men knowing about PCa and/or CRC. A higher percentage of older men (78%, *p* = 0.0027) knew that CRC home screening tests exist compared to only 22% of younger men knowing that there are CRC screening tests that can be done at home.

Regarding testing, older men were significantly more likely to be tested compared to younger men (*p* < 0.0001) which was consistent for AA, HA and AA/HA men. 56% of men were ever tested for PCa and CRC, while 44% of men report ever being tested for PCa. Among the AA men, older men were significantly more likely to have been tested for PCa and CRC (80%, *p* = 0.0022) than younger AA men (9%). The one older HA man was tested for PCa (*p* = 0.02) compared to younger aged HA men.

Regarding willingness, older men, regardless of race and ethnicity, were significantly more willing to buy/use a PCa home test (*p* = 0.01) or a CRC home test (*p* = 0.07) compared to younger men. Although, 56% of younger men were willing to buy/use a PCa or CRC at-home tests.

Younger men were statistically significantly more likely to be knowledgeable (OR = 0.08), have been tested (OR = 0.007) or willing (OR = 0.14) compared to older men (see Table 3). Although not statistically significant, trends indicate that AA/HA (OR = 0.17) and HA (OR = 0.33) are less likely to be tested for PCa or CRC compared with AA men.

### Barriers and desired resources for completing an at-home screening

**Figure 2.**
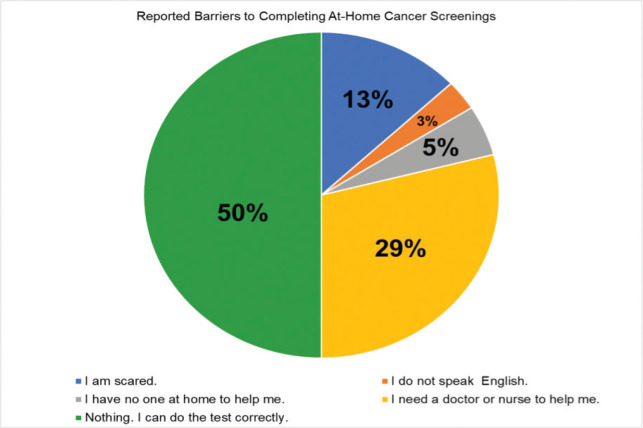
Barriers to completing at-home cancer screenings.

When examining all participants, 50% of participants believed that they could complete an at-home cancer screening test without assistance (see Figure 2). The most cited barrier was needing a doctor or nurse to help (29%), followed by being scared (13%). A total of 5% of participants reported not having anyone at home to help while 3% cited not speaking English. When stratifying by participants > 50 years old, 100% of AA/HA (*n* = 3) men reported at least one barrier. In particular, they reported being scared, needing a doctor or nurse to help them and not speaking English. Among AA men, 75% believed they could accurately complete an at-home test while one AA male reported needing a doctor or nurse to help. However, 100% of HA men (*n* = 1) reported being able to complete the at-home test without assistance.

**Figure 3.**
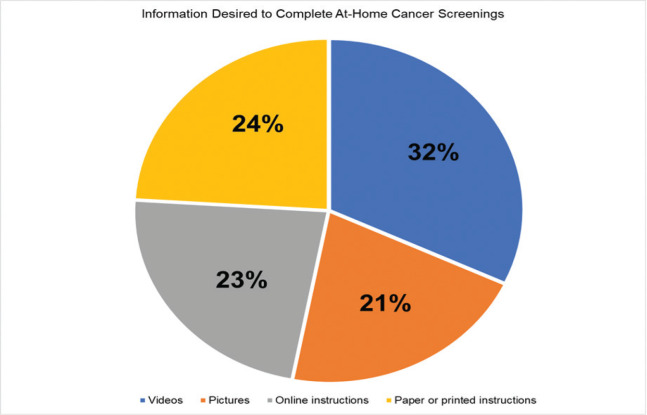
Information desired to complete at-home cancer screenings.

Regarding how one desires getting information (see Figure 3), receiving videos was preferred the most (32%), followed by paper or printed instructions (24%), online instructions (23%) and pictures (21%). However, among men > 50 years old wanted videos (37.5%), paper or printed instructions (37.5%) or some combination of picture, video or printed material (25%). No man > 50 years preferred to receive information via online instruction.

## Discussion

Findings from our study show that the majority of AA and/or HA men are aware of PCa and CRC, with 100% of men over 45 being aware of these two cancers. We also found that 44% of men aged > 45 years were screened for PCa, while the remaining 56% were screened for CRC as well. These results are higher than recent reports from Roberts *et al* [10] and others who report about half of Black men in their sample were screened for PCa. There are opportunities to improve knowledge since we found that a low percentage of younger men knew that there are at-home screening tests available for CRC, representing an opportunity for improved health education. We believe that the low knowledge level of at-home cancer screenings existing was in part due to a greater percentage of younger respondents, which would inherently affect the number of men who would have previously been screened for PCa and/or CRC. What is encouraging is that the majority of men in this study, except younger AA men, reported a willingness to perform at-home screening tests for PCa or CRC.

### Study strengths

The major strength of this study is learning more about knowledge, experiences and willingness of AA and/or HA men regarding PCa and colon cancer. Although our sample failed to have a high percentage of older men, there is still great significance in this study. First, it is important to begin exploring preferences of younger AA and or HA men who will eventually be screened for PCa or CRC; their knowledge levels, preferences and willingness for at-home CRC screening are relatively unknown. Second, another strength of this study is early exposure about PCa and CRC among AA and/or HA populations, groups currently at increased risk for developing and dying from PCa and CRC. Leading organisations like the ACS [1] recommend implementing programmes and initiatives that help younger aged men, especially those at risk, become aware and knowledgeable about cancers significantly affecting them.

Another strength of this research is the development of a questionnaire and resource guide that can be modelled or used by other researchers or healthcare professionals. Providing a resource guide to 100% of participants regardless of age is a study strength. The resource guide included facts, questions for providers and local screening locations, all of which increase participants’ awareness of PCa and colon cancers. Theoretically, providing information on PCa and CRC should increase knowledge about screening among AA and HA men. Participants being able to save and share the resource guide were strategically included to work as an additional effort to promote awareness and knowledge of these two cancers where participants will use this information as well as share with others.

### Study limitations

Our study, however, has potential limitations. For example, this was a cross-sectional study which used self-reported measures that are vulnerable to recall bias. Additionally, due to the 2019 novel coronavirus, our study was conducted exclusively online; therefore, results of our study might only reflect AA and HA men who are technologically savvy. Consequently, there were an unequal number of men who self-identified as being HA, AA or both HA and AA. Also, current limitations affected the age group of recruited Black males, since our study was digitised. Most men who participated in our study were below the recommended ages to screen for PCa and colon cancer. While one major limitation is that only 36 men participated, initial power calculations showed that having at least 32 men provided sufficient power to detect a large effect size (0.50) between AA and HA men. On the other hand, having a larger sample size might provide more power to detect even smaller to medium effect sizes. Our results yielded significant findings in bivariate analyses regarding testing and willingness to buy/use at-home screening tests for PCa and CRC.

Another limitation is that 75% of participants were below 45 years of age (the recommended age for screening of at-risk groups). Thus, low knowledge, willingness to be screened and eligibility levels may be due to younger men not beginning to have these conversations with their healthcare providers. Health professionals play a significant role in informing and encouraging men to be screened for PCa and colon cancer. It is important to note that studies involving at-risk Black men aged 18–40 have suggested that early conversations (e.g. with healthcare providers) may make the decision-making process for screening at an older age more effective [17, 18].

Another potential limitation is that the study was conducted online where participants completed the survey online and opted to received educational resources electronically. However, in view of the current pandemic, conducting research and providing healthcare information is increasingly being done via virtual platforms and in online environments, and is becoming more reflective of the general population of AA and HA men. Thus, this study also provides important information regarding the potential feasibility of conducting such studies with AA and HA men of various ages.

### Future research

Cancer research that has the HA population as the primary focus needs to be conducted. In addition to the number of scholarly and peer-reviewed articles focusing specifically on the subject of cancer in the HA populations are limited, which might serve to obscure cancer experiences such as incidence, preference and mortality in this population. More research on HA men cancer prevalence, mortality and knowledge needs to be explored. Involving HA men in research studies will introduce and potentially destigmatise conversations regarding cancer. This is increasingly relevant given findings from Pinheiro *et al* [6] showing that there is significant heterogeneity in cancer mortality between Blacks, HAs and men from other Caribbean islands (i.e. Jamaica).

Future research can also explore what AA or HA men specifically know about PCa and CRC, such as the various types of tests available, the potential accuracy and benefits of each test, and whether these tests can be performed at home. Our study only asked participants if they heard about PCa or CRC but did not ask participants to elaborate. To add, this research study took place during the 2019 novel coronavirus, as a result the popularity of telehealth and online health-services has increased. Future studies can examine whether HA men performing at-home cancer screenings have better outcomes compared to HA men who receive colonoscopies, or who forego screening altogether.

Other opportunities for future research are to replicate this study using an equal and greater number of AA, HA and AA/HA men for PCa and CRC. The methodology of the study, including the survey instrument, could be modified to assess a deeper level of knowledge regarding PCa and CRC screening. Additional studies can recruit focus groups to explore any family history of PCa or CRC which has been shown to influence knowledge and awareness in at-risk individuals. Focus groups will provide more freedom for participants to express their thoughts on the main ideas of this study.

## Conclusions

As at-home cancer testing options are becoming increasingly available, there is potential to increase screening options in Black male populations, a group at high risk for certain cancers. This research showed that Black men, identifying as HA and/or AA, are willing to use at-home tests. We also believe that initiatives to increase awareness and knowledge about PCa and CRC among younger AA and HA men might help substantially in increasing acceptance in this group who eventually will require PCa and CRC testing. All PCa and CRC materials being developed specifically to increase at-home PCa and CRC screening usage among AA and HA men (older or younger ages) should use videos and printed materials.

## Conflicts of interest

No one on the team has a conflict of interest.

## Authors' contributions

Alexandra Jean-Louis: Author of concept, literature search, protocol development and design, IRB and Scientific Review and Monitoring Committee (SMRC). approval, material development, study promotion and data collection, survey development and creation in Qualtrics, assisted with data analysis, led manuscript completion to include results, discussion and conclusions.

Fern J Webb, PhD: Protocol development and design, assistance with IRB & SMRC approval, guidance for resource development, data analysis and results, assisted with manuscript development and completion.

## Funding declaration

Research reported in this publication (or report) was supported by the National Cancer Institute, Center for Reducing Cancer Health Disparities of the National Institutes of Health under award numbers U54CA233396, U54CA233444 & U54CA233465, which support the Florida-California Cancer Research, Education and Engagement Health Equity Center. The content is solely the responsibility of the authors and does not necessarily represent the official views of the National Institutes of Health. The final peer-reviewed manuscript is subject to the National Institutes of Health Public Access Policy.

## Trial registration

This study was not registered with clinicaltrials.gov. This study was reviewed and approved by the UF IRB (#202001658) and the UF SMRC (#OCR37462).

## Figures and Tables

**Table 1.** Survey questions. Ten questions were developed for preliminary screening (1–3) and to gather PCa/CRC-related data (4–10).

Survey
1. Do you identify as an AA male? Yes No
2. Do you identify as a HA male? Yes No
3. Are you a male who is at least 18 years old? Yes No
4. Do you know what PCa and colon cancer are? Yes Only PCa Only colon cancer No
5. Have you ever tested for PCa and colon cancer? Yes Only PCa Only colon cancer No
6. There are colon cancer screening tests that can be done at home. Yes No
7. If a PCa screening test that can be done at home were made would you buy and use it? Yes No
8. If a colon cancer screening test that can be done at home were made would you buy and use it? Yes No
9. If any, select what will stop you from doing a cancer test correctly at home? Select all that apply. I am scared. I do not speak English. I cannot read English. I need a doctor or nurse to help me. I have no one at home to help me. I have no one at home who can read English. Nothing. I believe that I can do the test accurately. Other: [Will need to be specified]
10. What will help you do a cancer test correctly at home? Select all that apply. Videos Pictures Online instructions Paper or printed instructions Other: [Will need to be specified]

**Table 2.** Bivariate analyses of AA and/or HA men stratified by age ≥ 45 years versus <45 years.

	Total *N* (%)		AA/HA *N* (%)		AA *N* (%)		HA *N* (%)	
	≥45 years	<45 years	≥45 years	<45 years	≥45 years	<45 years	≥45 years	<45 years
**Knowledge**								
Do you know what PCa and colon cancer are? Yes Only PCa Only CRC Neither	*p* = 0.13		*p* = 0.39		*p* = 0.43		*p* = 0.57	
	9 (100) 0 0 0	18 (67) 5 (19) 0 4 (15)	3 (100) 0 0 0	7 (58) 2 (17) 0 3 (25)	8 (73) 2 (18) 0 1 (9)	5 (100) 0 0 0	1 (100) 0 0 0	3 (75) 1 (25) 0 0
There are colon cancer screening tests that can be done at home? Yes No	*p* = 0.0027		*p* = 0.17		*p* = 0.04		*p* = 0.02	
	7 (78) 2 (22)	6 (22) 21 (78)	2 (67) 1 (33)	3 (25) 9 (75)	4 (80) 1 (20)	3 (27) 8 (73)	1 (100) 0	0 4 (100)
**Current Testing**								
Have you ever tested for PCa or colon cancer? Yes Yes, only PCa Yes, only colon cancer No	*p* < 0.0001		*p* = 0.0006		*p* = 0.0022		*p* = 0.02	
	5 (56) 4 (44) 0 0	1 (4) 0 0 26 (94)	1 (33) 2 (67) 0 0	0 0 0 12 (100)	4 (80) 1 (20) 0 0	1 (9) 0 0 19 (91)	0 1 (100) 0 0	0 0 0 4 (100)
**Willingness**								
Buy/use a PCa screening test that can be done at home? Yes No	*p* = 0.01		*p* = 0.33		*p* = 0.01		*p* = 0.36	
	9 (100) 0	15 (56) 12 (44)	3 (100) 0	9 (75) 3 (25)	5 (100) 0	4 (36) 7 (64)	1 (100) 0	2 (50) 2 (50)
Buy/use a colon cancer screening test that can be done at home? Yes No	*p* = 0.07		*p* = 0.33		*p* = 0.10		*p* = 0.36	
	8 (89) 1 (11)	15 (56) 12 (44)	3 (100) 0	9 (75) 3 (25)	4 (80) 1 (20)	4 (36) 7 (64)	1 (100) 0	2 (50) 2 (50)

**Table 3.** Logistic regression modelling knowledge, testing and willingness to ethnicity and age.

	Knowledge OR (95% CI)	Willingness OR (95% CI)	Testing OR (95% CI)
Race/ethnicity AA/HA HA AA	0.79 (0.14–4.24) 0.32 (0.02–5.08) 1.00	5.25 (0.84–31.5) 1.75 (0.17–17.7) 1.00	0.17 (0.09–1.01) 0.33 (0.09–1.01) 1.00
Age <45 years old >45 years old	0.08 (0.13–0.52) 1.00	0.14 (0.00–0.99) 1.00	0.007 (0.00–0.94) 1.00
